# Influence of polymorphisms in TNF-α and IL1β on susceptibility to alcohol induced liver diseases and therapeutic potential of miR-124-3p impeding TNF-α/IL1β mediated multi-cellular signaling in liver microenvironment

**DOI:** 10.3389/fimmu.2023.1241755

**Published:** 2023-12-11

**Authors:** Debanjali Dasgupta, Suchandrima Ghosh, Indrashish Dey, Swagata Majumdar, Saheli Chowdhury, Subhas Das, Sanjana Banerjee, Mehelana Saha, Amit Ghosh, Neelanjana Roy, Alak Manna, Sukanta Ray, Shaleen Agarwal, Pradeep Bhaumik, Simanti Datta, Abhijit Chowdhury, Soma Banerjee

**Affiliations:** ^1^ Centre for Liver Research, School of Digestive and Liver Diseases, Institute of Post Graduate Medical Education and Research, Kolkata, India; ^2^ Department of Pharmacology, Institute of Post Graduate Medical Education and Research, Kolkata, India; ^3^ Department Gastro-Surgery, School of Digestive and Liver Diseases, Institute of Post Graduate Medical Education and Research, Kolkata, India; ^4^ Liver Transplant and Biliary Sciences, Max Saket West Super Speciality Hospital, New Delhi, India; ^5^ Department of Medicine, Agartala Government Medical College, West Tripura, India; ^6^ Department of Hepatology, School of Digestive and Liver Diseases, Institute of Post Graduate Medical Education and Research, Kolkata, India

**Keywords:** alcohol induced liver diseases, microRNA-124-3p, TNF-α, IL1β, alcohol, ALD, miRNA, liver microenvironment

## Abstract

**Background and aims:**

Alcoholic liver disease (ALD) is the leading cause of the liver cirrhosis related death worldwide. Excessive alcohol consumption resulting enhanced gut permeability which trigger sensitization of inflammatory cells to bacterial endotoxins and induces secretion of cytokines, chemokines leading to activation of stellate cells, neutrophil infiltration and hepatocyte injury followed by steatohepatitis, fibrosis and cirrhosis. But all chronic alcoholics are not susceptible to ALD. This study investigated the causes of differential immune responses among ALD patients and alcoholic controls (ALC) to identify genetic risk factors and assessed the therapeutic potential of a microRNA, miR-124-3p.

**Materials and methods:**

Bio-Plex Pro™ Human Chemokine analysis/qRT-PCR array was used for identification of deregulated immune genes. Sequencing/luciferase assay/ELISA detected and confirmed the polymorphisms. THP1 co-cultured with HepG2/LX2/HUVEC and apoptosis assay/qRT-PCR/neutrophil migration assay were employed as required.

**Results:**

The combined data analysis of the GSE143318/Bio-Plex Pro™ Human Chemokine array and qRT-PCR array revealed that six genes (TNFα/IL1β/IL8/MCP1/IL6/TGFβ) were commonly overexpressed in both serum/liver tissue of ALD-patients compared to ALC. The promoter sequence analysis of these 6 genes among ALD (n=322)/ALC (n=168) samples revealed that only two SNPs, rs361525(G/A) at -238 in TNF-α/rs1143627(C/T) at -31 in IL1β were independently associated with ALD respectively. To evaluate the functional implication of these SNPs on ALD development, the serum level of TNF-α/IL1β was verified and observed significantly higher in ALD patients with risk genotypes TNF-α-238GA/IL1β-31CT+TT than TNF-α-238GG/IL1β-31CC. The TNF-α/IL1β promoter Luciferase-reporter assays showed significantly elevated level of luciferase activities with risk genotypes -238AA/-31TT than -238GG/-31CC respectively. Furthermore, treatment of conditioned medium of TNF-α/IL1β over-expressed THP1 cells to HepG2/LX2/HUVEC cells independently showed enhanced level of ER stress and apoptosis in HepG2/increased TGFβ and collagen-I production by LX2/huge neutrophil infiltration through endothelial layer. However, restoration of miR-124-3p in THP1 attenuated such inter-cellular communications and hepatocyte damage/collagen production/neutrophil infiltration were prohibited. Target analysis/luciferase-reporter assays revealed that both TNF-α/IL1β were inhibited by miR-124-3p along with multiple genes from TLR4 signaling/apoptosis/fibrogenesis pathways including MYD88, TRAF3/TRADD, Caspase8/PDGFRA, TGFβR2/MCP1, and ICAM1 respectively.

**Conclusion:**

Thus, rs361525(G/A) in TNF-α and rs1143627(C/T) in IL1β gene may be used as early predictors of ALD susceptibility among East Indian population. Impeding overexpressed TNF-α/IL1β and various genes from associated immune response pathways, miR-124-3p exhibits robust therapeutic potential for ALD patients.

## 1 Introduction

Alcohol is one of the oldest environmental insults that causes chronic liver diseases with a wide spectrum of manifestations including steatosis or fatty liver to steatohepatitis, fibrosis and cirrhosis (1, 2). Although the early stages of ALD are reversible with cessation of alcohol, cirrhosis or severe alcoholic hepatitis often lead to death (3). Despite significant advancement in understanding the disease biology, lack of any early predictor for high-risk individuals and unavailability of FDA approved therapy for end-stage liver diseases, ALD is still a major risk of liver-related mortality worldwide.

ALD is associated with chronic inflammatory responses due to changes in intestinal microbiome composition and infiltration of pathogen associated molecular patterns (PAMPS) such as bacterial lipo-polysaccharides (LPS), bacterial DNA into the portal circulation. These activate Kupffer cells (KC) through pattern recognition receptor (PRRs), mostly TLR4 and TLR9 and produce pro-inflammatory cytokines such as tumour necrosis factor TNF-α, IL-1β, IL-6, GM-CSF and chemokines such as MCP-1, MIP-1α, etc. through NF-κβ (4, 5). Subsequently, intracellular signaling begins through both MyD88 dependent IRAK4/IRAK1-TRAF6-MAPK-NF-κβ-TNFα axis and MyD88 independent TRAM-TRIF-TRAF3/TRAF6-IRF3/NF-κβ pathways (6). This TNF-α signaling through TLR4 axis in alcoholic hepatitis has been markedly appreciated but anti-TNF-α therapy often associated with hepatotoxicity (7), and bacterial/fungal infection (8). Diverse attempts have been executed to develop a potential molecule inhibiting inflammatory responses to alcohol using mice models. Seminal studies with knock out mice of MCP1/IRF3/TLR4/caspase-I have revealed that these mice are protected from various stages of ALD including steatosis, steatohepatitis, and fibrosis as inflammasome dependent signaling in KCs is inhibited (9–11). Albeit, the IL1 receptor antagonist showed promising results (12, 13), further studies are required prior to its therapeutic application.

To our surprise, only 10%-30% of chronic alcoholics develop ALD upon chronic exposure to alcohol (>80grams/day for ≥ 10 years) implicating genetic variations could be one of the major determinants of the disease. Although the impact of allele specificity on the alcohol metabolism and anti-oxidant genes has widely been studied in ALD (14–18), immune genes are rarely explored (19–21). Allele-specific up surging of TNF-α (-308A>G, rs1800629) in various auto-immune diseases such as asthma, psoriasis, rheumatoid arthritis, ulcerative colitis and IL-1β (-31T>C, rs1143627) in tuberculosis have been documented (22–26).

In recent decades, the contribution of microRNAs (miRNAs) in physiological and pathological liver function; its potential as diagnostic marker and therapeutic target for liver disease management have been established (27). The role of deregulated miRNAs in hepatocytes and KCs in rodents fed with alcohol depicted MiR-125b, let7 and miR-21 restrict functions of TNF-α, IL6, and TLR4 pathways by blocking NF-κβ mediated IL10 production (28–30). Chunyan Ma et al. have reported that miR-124 attenuates immune signaling in alveolar epithelial cells by repressing multiple proteins in TLR6 signaling pathways including TLR6/MyD88/TRAF6 (31). In light of these findings, using clinical specimens, and *in vitro* experiments, this study was intended to assess the genetic makeup of deregulated immune genes considering alcoholic individuals with single ethnic group from India and its impact on the expression of genes to gain an insight into the pathogenesis of the disease. Subsequently, therapeutic potential of miR-124-3p was evaluated considering its binding sites on the 3’UTR of TNF-α/IL-1β/IL8/MCP1 along with the genes from TLR4 pathways MYD88/TRAF3/TRADD/TRAF6, fibrotic growth factor receptors TGFBR2/PDGFRA and leucocyte adhesion protein ICAM1.

## 2 Materials and methods

### 2.1 Inclusion of samples

A total of 490 consecutive subjects belonging to a single ethnic group (Bengali) with history of significant ethanol consumption (more than 80 g/day for more than 10 years) (16, 19) was enrolled for the study from the indoor and outdoor of Department of Hepatology at IPGME&R, Kolkata, East India and Department of Medicine at AGMC, Tripura, North-East India. Diagnosis of ALC (n=168) was based on ultra-sonographic evidence of normal liver with normal level of ALT/AST, while cirrhotic ALD patients (n=322) were diagnosed with portal hypertension, esophageal/gastric varices with/without ascites and steatohepatitis patients with fatty liver, elevated level of ALT but no histological sign of cirrhosis. Following the same criteria, 100 ALD patients were included in the validation cohort. Healthy individuals (n=10) having normal ALT/AST were also included.

Although samples were included from two different regions of India having similar ethnicity, both the groups exhibited same pattern of allelic distribution, hence samples were clubbed.

### 2.2 Exclusion of samples

Subjects from different ethnic background, positive for anti-HBsAg, Anti-HCV, or HIV seropositive, having co-morbidities like diabetes mellitus, and unwilling to comply with the study protocol were excluded.

### 2.3 Collection of blood

Blood (5ml) was collected from every participant in EDTA-vial and used for isolation of chromosomal DNA (32), PBMC and neutrophil using histopaque 1077 (Sigma). Demographic, clinical and biochemical data of non-alcoholic normal, ALC and ALD patients are presented in **Tables 1A** and **1B**.

**Table 1A:** Demographic, biochemical and clinical features of discovery cohort consist of ALC and ALD patients.

Variables	ALC (n=168)	ALD (n=322)	p value
Epidemiology			
Gender M:F	168:0	322:0	NA
Age, Years (Mean ± SD)	44.33 ± 10.700	45.52 ± 11.351	NA
Child-Pugh Score (A:B:C)	–	7.3 ± 0.5	–
Ascitis	0/70	106/322	NA
Alcohol consumption	12034.87 ± 3459.26	13688.16 ± 5621.66	NS
Liver stiffness Measurement (kPa) Mean ± SD	–	7.46 ± 3.45	–
Laboratory Results			
Total Bilirubin (mg/dl) (Median, Range)	1 (0.6-1.2)	3.75 (2.4-21.4)	<0.001
Albumin (g/dl) (Mean ± SD)	4.243 ± 0.812	3.075 ± 0.5396	<0.001
ALT (u/l) (Median, Range)	30.5 (17-42)	58 (26-748)	<0.001
AST(u/l) (Median, Range)	33 (19-44)	66 (19-1125)	<0.001
INR (Mean ± SD)	1.12 ± 0.3	1.84 ± 0.8	<0.01

p values obtained from student’s t test. NS, not significant, “-” not applicable.

ALT, Alkaline transaminase; AST, Aspartate transaminase; INR, International normalized ratio.

**Table 1B:** Demographic, biochemical and clinical features of normal and ALD patient whose liver tissues were collected.

Variables	Normal Liver (n=4)	ALD (n=4)
Epidemiology		
Gender M:F	4:0	4:0
Age, Years (Mean ± SD)	47.45 ± 13.22	48.75 ± 5.68
Child-Pugh Score (A:B:C)	-	0:1:3
Ascitis	0/4	4/4
Alcohol consumption	No	Yes
Laboratory Results		
Total Bilirubin (mg/dl) (Median, Range)	0.6 (0.6-1.1)	8.75 (2-14.4)
Albumin (g/dl) (Mean ± SD)	3.90 ± 0.48	2.575 ± 0.53
ALT (u/l) (Median, Range)	39 (13-44)	32.5 (20-58)
AST(u/l) (Median, Range)	39 (16-44)	37.5 (16-67)
INR (Mean ± SD)	1.04 ± 0.11	1.93 ± 0.21

### 2.4 Non-alcoholic steatohepatitis

Non-alcoholic steatohepatitis (NASH) patients (n=50) with comparable age, sex and having no other liver diseases as verified histologically were included from the Department of Hepatology, IPGME&R, Kolkata, India (**Supplementary Table 1**).

### 2.5 Collection of liver tissues

Explanted liver tissues were collected from patients undergone orthotopic liver transplantation due to alcoholic cirrhosis (n=4) from Centre for Liver and Biliary Sciences, Indraprastha Apollo Hospital, New Delhi, India and confirmed with histological, and radiological evidences.

Normal liver tissues (n=10) were collected from gastrointestinal surgery clinic of IPGME&R, Kolkata, during cholecystectomy of gall bladder carcinoma (GBC) patients as routine analysis to test liver metastasis. Liver tissues with normal architecture (n=4) were included in the study after histological verification. Tissues were collected in RNA later (ThermoFisher, Cat. #AM7020) and preserved at -80^0^C for future use.

### 2.6 Transcriptome data analysis

RNAseq data of alcoholic hepatitis (n=5) vs. normal liver tissue (n=5) (GSE143318) was retrieved from https://www.ncbi.nlm.nih.gov/geo/query/acc.cgi?acc=GSE143318 and the data was analyzed using DEseq2 package to obtain differential gene expression (DEG). DEG with log_2_fold change>2 and P_adjusted_ value <0.05 were considered for further pathway analysis using Kyoto Encyclopedia of Genes and Genomes (KEGG).

### 2.7 Bio-Plex Pro™ Human chemokine analysis

A BioPlex ProHuman chemokine Screening Panel, 48 plex (Bio-Rad, # 120072831) was used to detect the levels of forty serum chemokines following the manufacturer’s protocol at National Institute of Biomedical Genomics, Kalyani, India. The data were analyzed using BioPlex® 200 Multiplexing Platform. DGE were performed as mentioned earlier and subjected to KEGG pathway analysis.

### 2.8 Identification of transcription factors binding to the promoter

The total promoter sequence of TNF-α and IL1ß were obtained from the University of California Santa Cruz (UCSC) Genome Browser, selecting the Human GRCh37/hg19 genome assembly. The entire promoter sequence was scanned using TFBIND tool for the transcription factor binding site. ChIP Seq (https://genome.ucsc.edu/) data was used for validation.

### 2.9 RNA isolation, reverse transcription and quantitative real-time PCR

Liver tissue (1mg) was homogenized and RNA was isolated using Trizol (ThermoFisher, # 15596026). About 2.5μg/500 ng of total RNA was used to synthesize cDNA for mRNA/miRNA using Revertaid Reverse Transcriptase (ThermoFisher, #MAN0012885)/miSCRIPT PCR starter kit (Qiagen, #218193) respectively. Expression of immune related genes was assessed in ABI QuantStudio 7 real-time PCR machine using SYBR green (Roche). Only upregulated genes in each sample were further considered for genotyping. Primers used for quantification of mRNAs and miRNAs are presented in **Supplementary Table 2A**.

### 2.10 Chromosomal DNA preparation and method of genotyping

Chromosomal DNA was isolated using salting out method from 3ml of EDTA-blood (14, 32). Variations in the promoter région was determined by sequencing with Big-Dye Terminator v3.1 (ThermoFisher, #4337455) on ABI Prism 3100 Genetic Analyzer followed by multi-sequence alignment using Sequencher software.

### 2.11 Cell culture, plasmid information, mutagenesis, transfection and luciferase assay

HepG2 (Hepatoblastoma), and LX2 (Stellate cell) cell lines were maintained in DMEM (HiMedia # AL067) with 10% and 2% FBS respectively while THP1 (monocytic cell line) was maintained in RPMI with 10% FBS. Endothelial cell growth media (Cell applications Inc.#211-500) was used for HUVEC (primary Endothelial cell line, Invitrogen). Cells were maintained at 37°C incubator with 5% CO_2_.

The full-length (FL) TNF-α cDNA was cloned in pcDNA3.1(+)/myc-HisB vector (ThermoFisher). Dr. Susanta Roychowdhury, Indian Institute of Chemical Biology, Kolkata, India gifted us two plasmids carrying FL-cDNA of the IL1β gene with promoter having -31CC/TT (pMC-31CIL-1β/pMC-31TIL-1β) (33). Premir-124 was cloned in pRNAT-U6.1/neo vector and pSiCHECK-2 dual-luciferase reporter vector was used for the cloning of 3’UTR region of each gene. The site-directed mutagenesis (SDM) kit (NEB) was used to generate miRNA binding site mutant following manufacturer’s protocol. Primers for 3’UTR cloning and SDM are presented in **Supplementary Tables 2B, C**.

Lipofectamine 2000 (ThermoFisher, #11668019) and x-tremeGENE HP transfection reagents (Sigma, #6366236001) were used for transfection in different cell lines as required. THP1 cells were treated with LPS (100ng/ml) for 12 hours after transfection. HepG2/THP1/LX2/HUVEC were either directly transfected with required plasmids or incubated with conditioned media (CM) of transfected THP1 cells (1:1 ratio) for 48 hrs. Each experiment was performed in duplicate and repeated three times.

### 2.12 ELISA of secreted cytokines

Secreted levels of TNF-α/IL1β in the serum of ALD/ALC individuals, and in cell culture supernatant were determined using ELISA following the manufacturer’s protocol (Ray BioTech, #P01375 and Abclonal, #RK00001).

### 2.13 LPS treatment of PBMC

About 2x10^6^ PBMCs were seeded on 6 well plates. After 24 hours, cells were either stimulated with LPS (100ng/ml) for 6 hours or left untreated and subsequently total RNA was isolated.

### 2.14 Apoptosis assay

Apoptotic cells were quantified in BD-FACS VERSE using Annexin V and PI staining kit (Invitrogen, #556547) after 48 hours of transfection following manufacturers’ protocol. Early (Annexin v-FITC^+^) and late apoptotic cells (Annexin V-FITC^+^PI^+^) were considered for quantification of total apoptotic cells.

### 2.15 Trans-endothelial migration assay

HUVEC cells (5x10^4^ cells/well) were seeded on the collagen pre-coated upper well of the Boyden Chamber (Cell BioLabs, 3μm pore size, #CBA-103) and either directly transfected or conditioned media (CM) of pTNF-α/Anti-TNF-α anti-miR-124-3p oligo transfected in THP1 cells (1:1) were added. The lower chamber was filled with 30% FBS containing media and freshly isolated neutrophil (10^6^ cells) was added on the upper chamber and number of cells migrated to the lower chamber was counted at different time points (2-24 hrs) using hemocytometer.

### 2.16 Statistical analysis

All the statistical tests were performed using SPSS version 10.0 and Graphpad prism. Hardy-Weinberg Equilibrium test was done for each SNP to determine whether the SNP present in this population is in equilibrium or influenced by any evolutionary forces. The association of genotype and allele frequencies were calculated using Pearson’s chi-square test (14, 19). Unpaired two tailed Student’s t-test was performed for comparison of expression data. T-test and Mann Whitney U test was employed for biochemical parameters as required. The parameters were presented either as mean ± SD (for normally distributed parameters) or as median and minimum to maximum distribution with range of the datasets (for skewed distributed parameters). Individual predictors were determined using multiple logistic regression analysis with significantly associated SNPs in Univariate analysis. P-values were corrected using Benjamini−Hochberg multiple testing correction. P_adjusted_ <0.05 was considered as significant.

## 3 Results

### 3.1 Demographic, clinical and biochemical characteristics of the study subjects

This study has included 490 individuals in discovery cohort (ALD=322 and ALC=168) and 150 individual in validation cohort (ALD=100 and NASH=50). In addition, 10 healthy individuals were incorporated. Explanted Liver tissues were also collected from four ALD patients. Liver biopsies from gall bladder carcinoma patient (n=10) with normal histology were considered as normal (n=4).

### 3.2 Selection of overexpressed ALD susceptible immune genes

**Figure 1:**
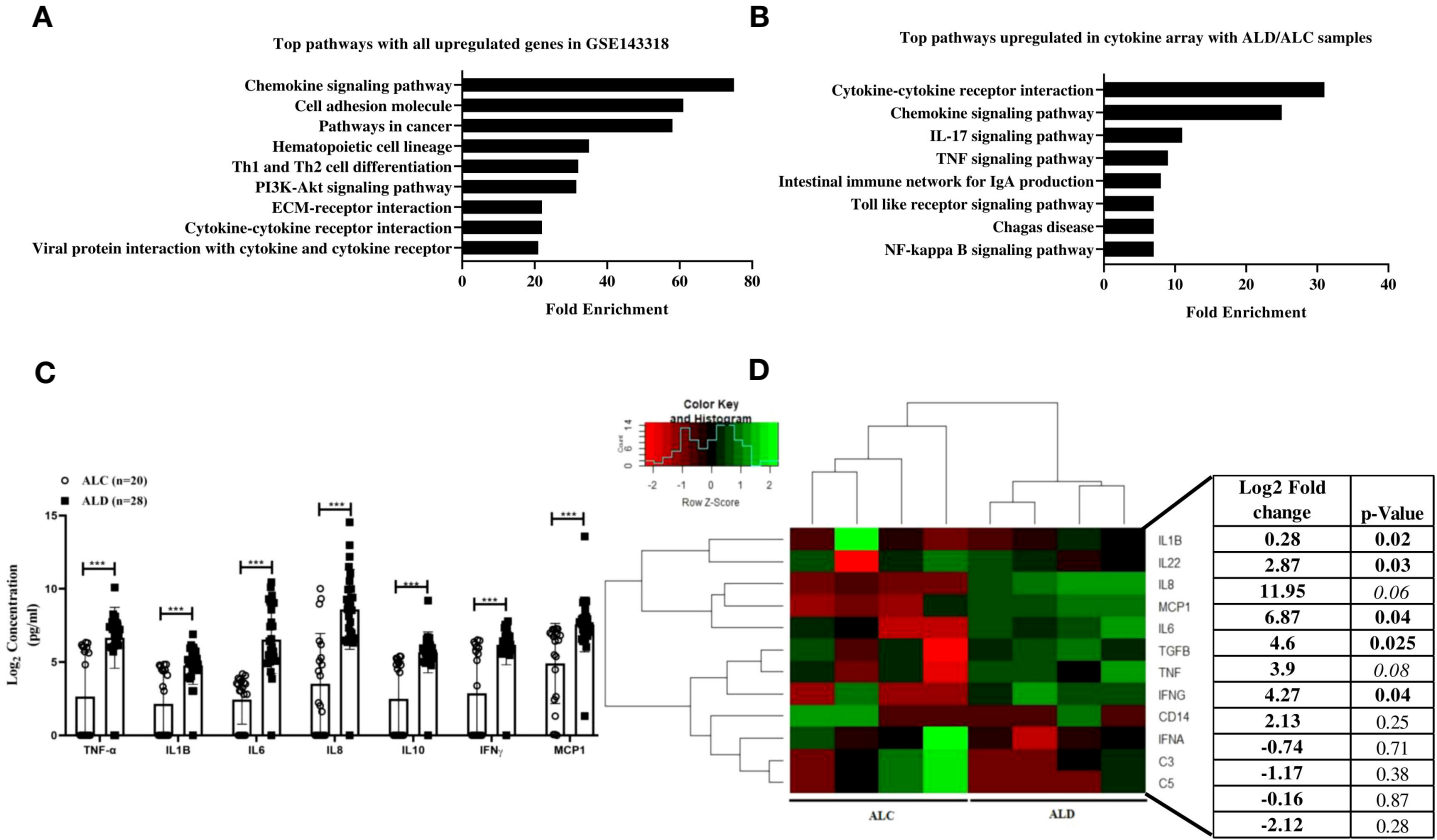
**(A)** GSE 143318 data was analyzed using DEseq2 packages to identify differentially expressed genes in ALD (n=5) vs. Control (n=5) liver tissue samples and KEGG pathway analysis was performed with significantly upregulated genes. **(B)** KEGG pathway analysis with differential gene expression data obtained from BioPlex ProHuman chemokine Panel assay using serum sample of Alcoholic control (ALC, n=20) and Alcoholic liver disease (ALD, n=28) patients, **(C)** Presentation of commonly altered genes in serum and liver tissue **(D)** Unsupervised hierarchical heatmap with qRT-PCR array data performed with liver tissue samples from ALC and ALD patients. p<0.05 was considered significant and *** means p<0.001.

Despite having similar amount of alcohol consumption, all alcoholics are not susceptible to ALD suggesting genetic variations play crucial role here. To identify the genes responsible for this differential behavior, overexpressed genes in ALD were identified after analysis of the transcriptome data, GSE 143318 (Alcoholic hepatitis vs. Control). It was followed by KEGG pathway analysis and revealed that the chemokine signaling pathways were the most upregulated pathways in ALD (**Figure 1A**). Furthermore, our Bio-Plex Pro™ Human Chemokine Panel, 40-Plex assay with serum of ALD (n=28) and ALC (n=20) individuals also disclosed cytokine-cytokine receptor signaling and chemokine signaling pathways were the highest upregulated pathways in ALD (**Figure 1B**). Thus, we focused on upregulated immune genes only and genes were selected after validation in the liver tissue of ALD (n=4) and ALC (n=4) using customized qRT-PCR expression array which included immune genes associated to alcohol induced steatohepatitis and cirrhosis such as TLR4, IFN-α, CD14, MCP1, IL8, TGF-β, TNF-α, IL6, IL18, IFN-γ, IL22, IL17, IL10, CCR2 and complements C5 and C3 as found in the published literatures (19, 34, 35) and in the above two analyses. After comparative analysis of the above two arrays, six genes TNF-α, IL6, MCP1, IL1β (P value<0.05) and TGF-β, IL8 (border line significant in liver tissue) were found up-regulated in both the serum and liver tissue samples of all the ALD patients tested compared to ALC (**Figures 1C, D**), which suggested their significant contribution in the ALD development.

### 3.3 Association of SNPs in the immune genes with ALD

Now, to find the reason of ALD development in only 10-30% of chronic alcoholics, the serum cytokine data of all the six genes in ALC group was carefully investigated (**Figure 1C**) and observed that this group had significantly lower level of serum cytokines albeit they were exposed to the same volume of alcohol compared to the ALD patients. A group of ALC individuals were noted with cytokines level below the detection limit. To understand this disparity in expression, the entire promoter regions of all the above six genes were sequenced in 322 ALD and 168 ALC individuals and noticed no sequence variations in MCP1, IL6, TGF-β and IL8 promoters. But, three SNPs, rs361525 (G/A) at -238 of TNF-α promoter and rs1143627(C/T) at -31 & rs16944(C/T) at -511 in IL1β promoter were noted and the frequencies were more in ALD individuals than ALCs (15.9% vs.3%, p<0.01; 65.5% vs. 40%, p<0.01; 48% vs. 61.8%, p<0.01 respectively) (**Table 2A**). Further univariate analysis showed significant association of all the three SNPs with ALD but multivariate analysis revealed that rs361525G/A [(OR 1.75, 95% CI (1.22-2.76)] and rs1143627/C/T+TT (OR 2.32, 95% CI (1.78-3.62) were two independent risk alleles which might confer susceptibility to ALD (**Table 2B**). This data was again validated in a new cohort of 100 ALD patients and 50 NASH patients and insignificant association was observed with NASH patients (Data not shown).

**Table 2A:** Allele frequency of rs361525 in TNF-α and rs1143627, rs16944 in IL1β among the study population.

Gene name	Locus ID	Allele	Allele frequency		p- value
			ALC	ALD	
TNFα	rs361525	G/G G/A A/A	n= 168	n= 277	Ref **<0.01** NA
			161 (0.96) 5 (0.03) 0 (0)	233 (0.84) 43 (0.16) 1 (0.004)	
IL1β	rs1143627	C/C C/T T/T	n=168	n=220	Ref **<0.01** NA
			106 (0.63) 61 (0.36) 1 (0.006)	76 (0.35) 106 (0.48) 38 (0.17)	
IL1β	rs16944	C/C C/T T/T	n=168	n=220	Ref ns **0.018**
			87 (0.52) 30 (0.18) 51 (0.30)	84 (0.38) 34 (0.15) 102 (0.46)	

P value<0.05 means significant.

**Table 2B:** Univariate and Multiple logistic regression analysis to determine the independent risk factors for development of Alcoholic liver diseases.

Gene name	Loci id	OR, 95% CI (Univariate analysis)	p value (Univariate analysis)	OR, 95% CI (Multivariate analysis)	p value (Multivariate analysis)
TNF-α	rs361525	2.88 (2.22-3.41)	<0.01	1.75 (1.22-2.76)	**0.018**
IL1β	rs1143627	2.28 (1.92-3.62)	<0.001	2.32 (1.78-3.62)	**0.006**
IL1β	rs16944	1.48 (1.08-2.18)	<0.05	1.24 (0.98-2.41)	*0.062*

P value<0.05 means significant.

### 3.4 Impact of SNPs on the expression of TNF-α and IL1β genes in ALD Patients

**Figure 2:**
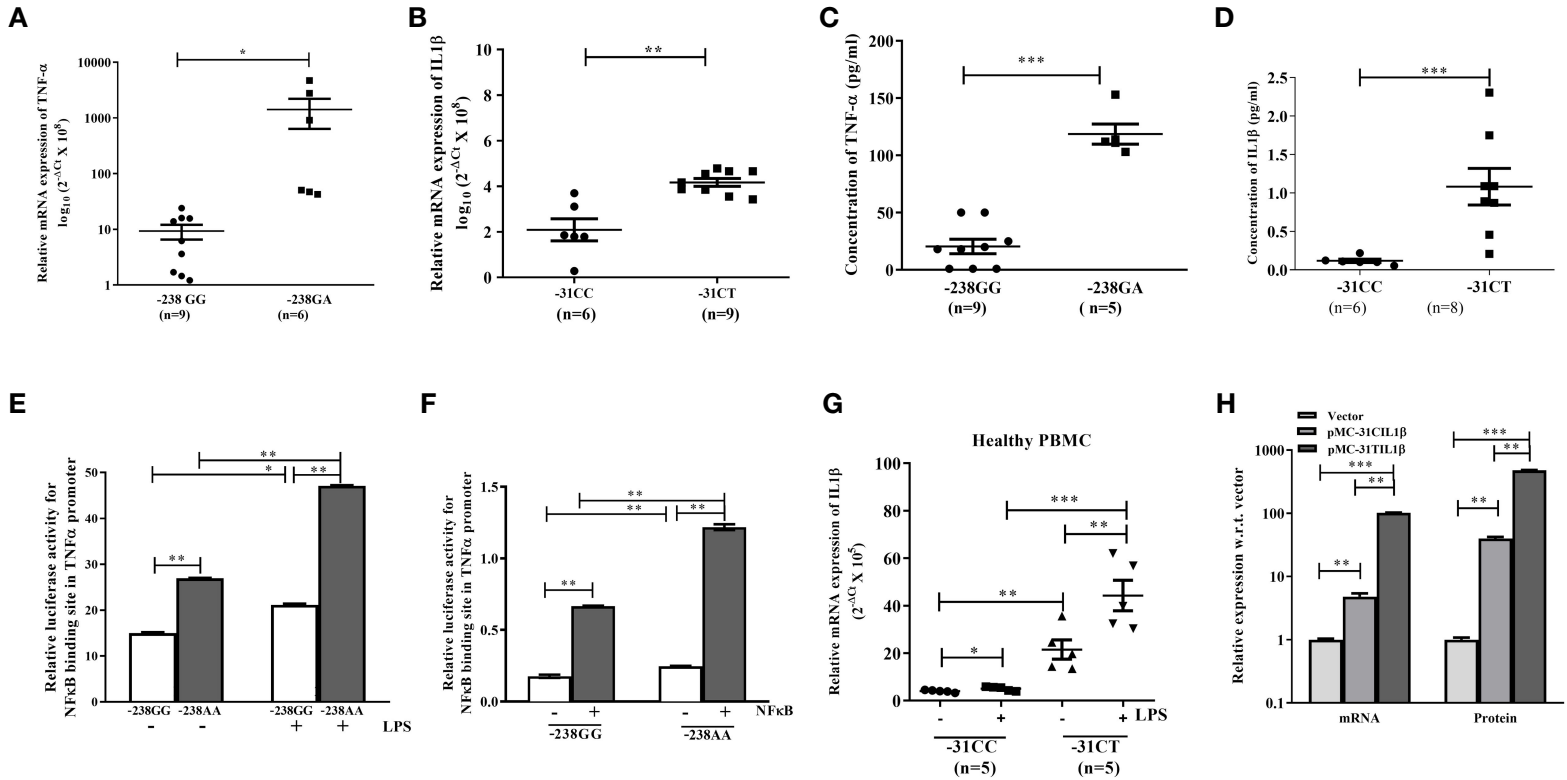
**(A–D)** qRT-PCR analysis and ELISA with liver tissue and serum of normal individuals having TNF-α with rs361525(GG/GA) at -238 and IL1β with rs1143627(CC/CT) at -31. **(E, F)** Luciferase assay with TNF-α promoter having either -238GG or -238AA in absence or presence of LPS and NF-κβ. Quantification of IL1β with -31CC and -31TT genotype using **(G)** PBMC isolated from normal individual, and **(H)** pIL1β-31CC and pIL1β-31TT transfected in HepG2 cells. *,**,*** indicate p<0.05, 0.01 and 0.001.

Now, to determine the impact of two SNPs on the expression of TNF-α and IL1β, normal individuals carrying the above SNPs were tested by qRT-PCR and ELISA. To our surprise, the expression of TNF-α and IL1β were noted significantly higher in the liver tissue of the normal individuals carrying rs361525GA at -238 (n=6) than GG (n=9) and rs1143627CT at -31(n=9) than CC (n=6) genotype respectively (**Figures 2A, B**). ELISA data of normal individuals with risk allele rs361525GA and rs1143627CT also showed higher level of TNF-α and IL1β than GG and CC allele respectively (**Figures 2C, D**). These data suggest that aforementioned SNPs in the promoter of TNF-α and IL1β might be contributed to the disease pathogenesis in susceptible individuals.

Next, to understand the mechanism of overexpression of TNF-α and IL1β in susceptible individual, UCSC-ChIP-seq data was analyzed and observed a NFκβ binding site at -239 position in the TNF-α promoter. Thus, Luciferase reporter assay with the TNF-α promoter constructs carrying either -238GG or -238AA genotype was performed in presence or absence of LPS and p65-NFκβ subunit. It was observed that the genotype “AA” showed higher luciferase activity than GG (**Figures 2E, F**) indicating that the presence of “A” at -238 loci might enhance the binding affinity of NFκβ to that site. Similarly, PBMC isolated from normal individual having either -31C/C or -31C/T allele in IL1β and subsequent qRT-PCR data depicted that the individual with -31C/T allele had significantly higher expression of IL1β than -31C/C at IL1β loci (T/T allele was absent in this population), in both presence or absence of LPS (**Figure 2G**). Next, when HepG2 cells were transfected with either pIL1β-31TT or pIL1β-31CC plasmids, the expression of IL1β was noted higher in presence pIL1β-31TT than pIL1β-31CC by qRTPCR and ELISA as well (**Figure 2H**). It is worthy to be noted that a new TATA box appears when the C allele replaces with T allele at -31 loci of IL1β promoter. So, binding of more TATA box binding protein plausibly induced the expression of IL1β in CT genotype carrying individuals.

In 2000, Hill et al, have reported that alcohol/LPS stimulates the expression of the nuclear factor NFκβ, which induces synthesis of TNF-α (36) and TNF-α and IL1β trigger a wide range of intracellular signaling pathways, which require IL6, MCP1, ICAM1 and TGF-β. To our surprise, multiple NFκβ binding sites were noted in the promoter regions of all these six genes which were significantly altered in our cohort (**Supplementary Figure 1A**). The data was validated in THP1 cells treated with LPS and observed overexpression of TNF-α, IL1β, MCP1, IL8 and NFκβ while the expression of each gene was also synergistically increased upon overexpression of TNF-α in HepG2 cells treated with LPS (**Supplementary Figures 1B, C**).

Thus, our data suggest that LPS induces expression of TNF-α and/or IL1β and individual carrying either rs361525G/A in TNF-α or rs1143627C/T in IL1β produces excessive amount of TNF-α and IL1β in the liver milieu. Now, TNF-α signaling through TLR4 pathway activates multiple intracellular as well as intercellular signaling cascades that lead to progression of liver diseases from steatosis to steatohepatitis, fibrosis and cirrhosis (13).

### 3.5 Selection of miR-124-3p that targets both TNF-α and IL1β

In purpose to abolish the effect of increased level of TNF-α and IL1β upon alcohol exposure, extensive bioinformatics analysis was performed using Targetscan, miRDB and miRwalk to select a common miRNA targeting both the genes. Here, a few more selection criteria were employed such as the selected miRNA was not abundant in the liver, and it was unaltered upon alcohol exposure. Following above criteria, miR-124-3p was selected which was an anti-inflammatory miRNA. The read count of miR-124-3p was evaluated from next generation sequencing data of ALD vs. normal liver tissue and it was quite low (Read Count<5, data not shown).

**Figure 3:**
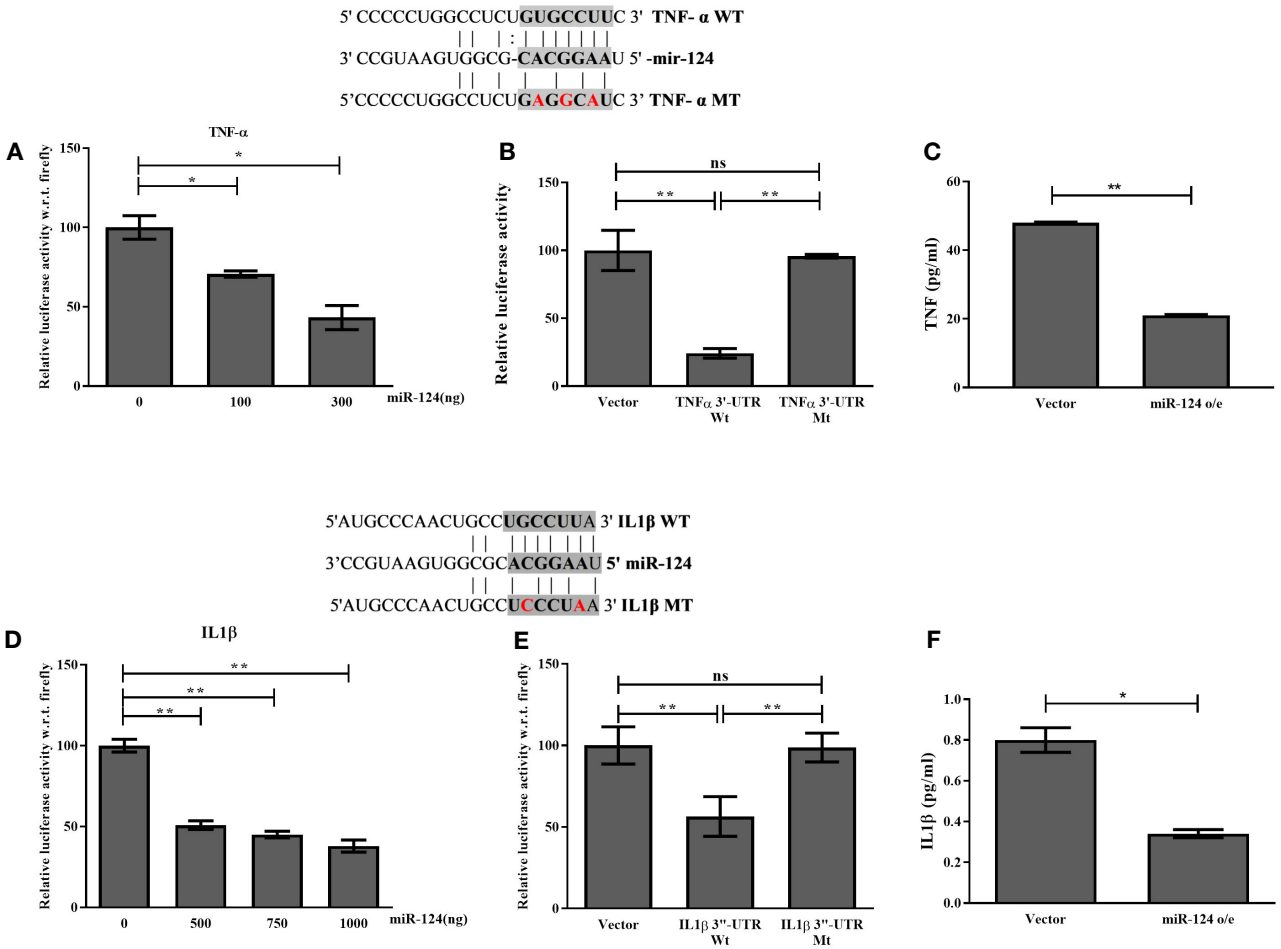
Luciferase assay with HepG2 cells co-transfected with **(A, D)** increasing doses of -pre-miR-124 and 3’UTR luciferase construct of TNF-α & IL1β, **(B, E)** -pre-miR-124 and 3’UTR luciferase wild type and mutant construct of TNF-α & IL1β. **(C, F)** ELISA of TNF-α & IL1β with media of HepG2 cells transfected with -pre-miR-124. *, and ** indicate p<0.05, and 0.01 and ns means not significant.

Next, the data obtained in bioinformatics analysis was validated by 3’-UTR-luciferase reporter assays using both wildtype (WT) and miR binding site mutant (MT) 3’UTR-Luciferase constructs. Thus, HepG2 cells were co-transfected with premiR-124 and WT/MT 3’UTR-luciferase constructs of TNF-α and IL1β. After 48h of transfection, a significant reduction in the luciferase activity was observed with WT construct and with increasing doses of miR-124-3p while the MT-luciferase constructs showed no response to miR-124-3p. Furthermore, ELISA data with the conditioned media (CM) of miR-124-3p overexpressed HepG2 cells depicted that the restoration of miR-124-3p in HepG2 suppressed the level of TNF-α and IL1β by 2.5 folds and 4 folds respectively (**Figures 3A–F**). So, these data clearly suggested that the binding of miR-124-3p to the 3’UTR of TNF-α and IL1β.

### 3.6 Effect of miR-124-3p on TNF-α and IL1β induced ER stress and apoptotic cell death

**Figure 4:**
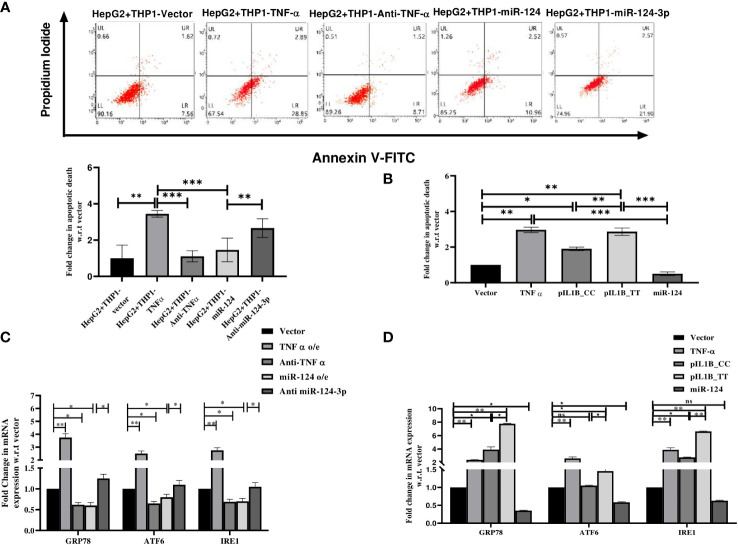
Apoptosis assay with **(A)** HepG2 cells treated with conditioned media of LPS treated THP1 cells transfected with control vector, TNF-α, anti- TNF-α, -pre-miR-124 and anti-miR-124-3p and **(B)** HepG2 cell directly transfected with TNFα, pIL1β-31CC, pIL1β-31TT, and -pre-miR-124. **(C, D)** qRT-PCR analysis with endoplasmic reticulum stress markers using similar setting of transfection. *,**,*** indicate p value <0.05, 0.01 and 0.001. ns means not significant.

Upon alcohol consumption, the gut derived bacterial endotoxin LPS mediates inflammation through CD14 and TLR4 to initiate MyD88 dependent and TRIF signaling cascade to activate NF-κB which regulates TNF-α and IL1β secretion from activated Kupffer Cells (KCs) (11). TNF-α binds to the TNF-α receptor (TNFR1) on the hepatocytes and activates TNFα receptor-associated death domain (TRADD) mediated extrinsic apoptosis pathway (37). Here, HepG2 cells were incubated with the CM of the LPS treated THP1 cells transfected with pTNF-α/Anti-TNF-α oligo/pre-miR-124/anti-miR-124-3p and apoptotic death of HepG2 cells was quantified in FACS acquiring AnnexinV-FITC^+^/AnnexinV-FITC^+^PI^+^ cells. Apoptotic cell death was noted higher in presence of TNF-α compared to the mock while it was reduced upon treatment with anti-TNF-α/miR-124 and anti-miR-124-3p treatment showed opposite data (**Figure 4A**). This experiment was also repeated in HepG2 cells directly transfected with the cDNA of TNF-α/pIL1β-CC(low IL1β)/pIL1β-TT(high IL1β)/pre-miR-124 independently in presence of LPS and observed higher apoptosis level upon overexpression of both TNFα, and pIL1β-TT than mock/pIL1β-CC and restoration of miR-124 in the milieu also suppressed the apoptotic death (**Figure 4B**).

Apart from TNFR1 mediated cell death, Alcohol-induced endoplasmic reticulum (ER) stress is now established as an important contributor to apoptotic cell death in ALD (38). Thus, expression of major ER chaperon protein GRP78, two transmembrane signaling molecules ATF6 and IRE1 were quantified in HepG2 cells treated with the CM of TNF-α cDNA, Anti-TNF-α, pre-miR-124, anti-miR-124-3p transfected THP1 cells or HepG2 cells were directly transfected with cDNA of TNF-α, pIL1β-CC(low IL1β)/pIL1β-TT(high IL1β) and pre-miR-124. All the ER stress markers were significantly overexpressed in the presence of TNF-α and pIL1β-TT compared to the vector and pIL1β-CC which were dropped upon treatment with either anti-TNF-α or miR124 and again re-established upon anti-miR-124-3p treatment (**Figures 4C, D**).

### 3.7 Effect of miR-124-3p on TNF-α and IL1β mediated liver fibrosis progression

**Figure 5:**
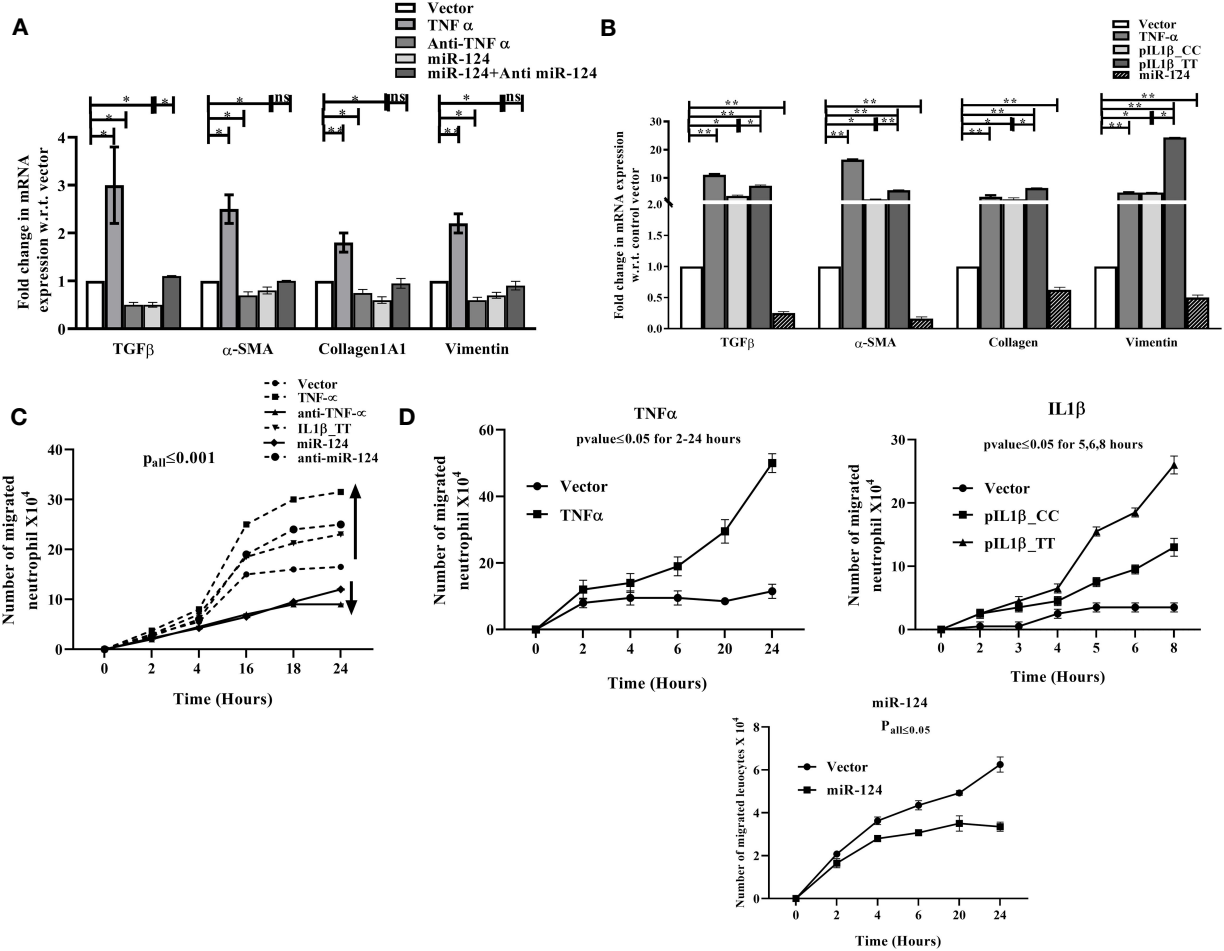
qRT-PCR analysis of fibrosis markers in **(A)** HepG2 cells treated with conditioned media of LPS treated THP1 cells transfected with control vector, TNF-α, anti- TNF-α, -pre-miR-124, anti-miR-124-3p and **(B)** HepG2 cells directly transfected with TNF-α, pIL1β-31CC, pIL1β-31TT, and -pre-miR-124. **(C, D)** Neutrophil infiltration assay with similar setting of transfection as **(A, B)** through HUVEC layer. HUVEC cells were seeded on upper well of Boyden chamber and either CM of THP1 transfected as mentioned in **(A)** or directly transfected with same setting as **(B)** and then fresh neutrophil was added. Infiltrated neutrophils were counted from lower chamber at different intervals. *,** indicate p value <0.05, 0.01. ns means not significant.

ALD patients are also associated with a significant amount of liver fibrosis. Upon ethanol exposure cytokines and profibrogenic factor TGF-β released from KCs and hepatocytes, which activate quiescent hepatic stellate cells (HSCs) and transform into myofibroblast-like cells. Activated HSCs secrete collagen and extracellular matrix (ECM), and fibrosis progresses (39). In our *in vitro* setting either CM of TNF-α cDNA, Anti-TNF-α, pre-miR-124, Anti-miR-124-3p transfected THP1 cells was added to HSCs or HSCs were directly transfected with cDNA of TNF-α, pIL1β-CC (low IL1β)/pIL1β-TT (high IL1β) and pre-miR-124. The expression of pro-fibrogenic factor TGF-β1, myofibroblast markers Vimentin, α-SMA and Collagen1A1 was induced in presence of TNF-α or IL1β, which was suppressed upon anti-TNF-α and miR-124 treatment and regained upon restoration of Anti-miR-124-3p (**Figures 5A, B**).

### 3.8 Effect of miR-124-3p on TNF-α and IL1β induced neutrophil infiltration through endothelial barrier

Neutrophil infiltration in the liver is a hallmark of alcoholic hepatitis. The chemokines/cytokines such as IL8, MCP1, MIP2 etc. released by the LPS exposed KCs behave as chemotactic molecule for neutrophils to migrate to the site of inflammation (40). Thus, to mimic the milieu, HUVEC cells seeded on the collagen-coated upper well of Boyden chamber was treated with the CM of TNF-α cDNA/Anti-TNF-α/pre-miR-124/Anti-miR-124-3p transfected THP1 cells or directly transfected with cDNA of TNF-α/pIL1β-CC (low IL1β)/pIL1β-TT (high IL1β)/pre-miR-124 and freshly isolated neutrophils were added on top of it. Infiltrated neutrophils were counted in the lower chamber and found higher in TNF-α/IL1β overexpressed condition than the controls which was decreased upon treatment with anti TNF-α/miR-124 and reversed when treated with Anti-miR-124-3p (**Figure 5C**). Similar data was obtained upon direct transfection of plasmids in HUVEC cells (**Figure 5D**).

### 3.9 Mechanism of function of miRNA-124-3p

**Figure 6:**
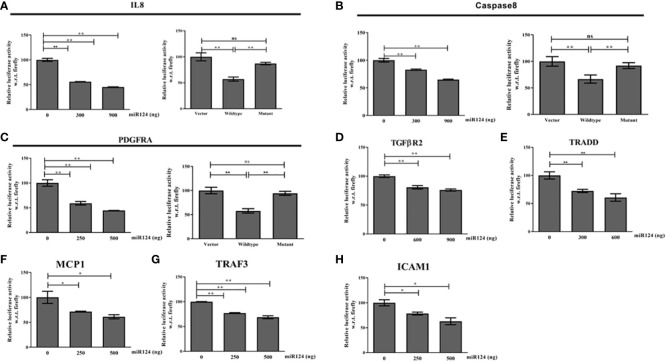
**(A–H)** 3’UTR luciferase reporter assay of IL8, Caspase8,PDGFRA, TGFBR2, TRADD, MCP1,TRAF3, and ICAM1 with increasing doses of miR-124-3p. Luciferase assay was performed with wild type and mutant 3’UTR luciferase construct of IL8, Caspase8 and PDGFRA as we are showing for the first time. *,** indicate p value <0.05, 0.01 and ns means not significant.

Next to understand the mechanism of function of miR-124-3p, the other target genes from TLR4 signaling axes were retrieved using Bioinformatics analysis. It is noteworthy to be mentioned that miR-124-3p was found to target various genes involved in LPS mediated inflammatory signaling pathways such as MyD88, TRAF6 and TRAF3; TRADD, Caspase8; IL8, MCP1, ICAM1 and TGFBR2 and PDGFRA (**Supplementary Table 3**). The data was validated by 3’-UTR luciferase assays with WT/MT luciferase constructs of each target gene and the data has confirmed the binding of miR-124-3p to TRADD, Caspase8, PDGFRA, TGFβR2, IL8, ICAM1, and MCP1 (**Figure 6**). Binding of miR-124-3p to the 3’UTR of other genes has been reported (31).

**Figure 7:**
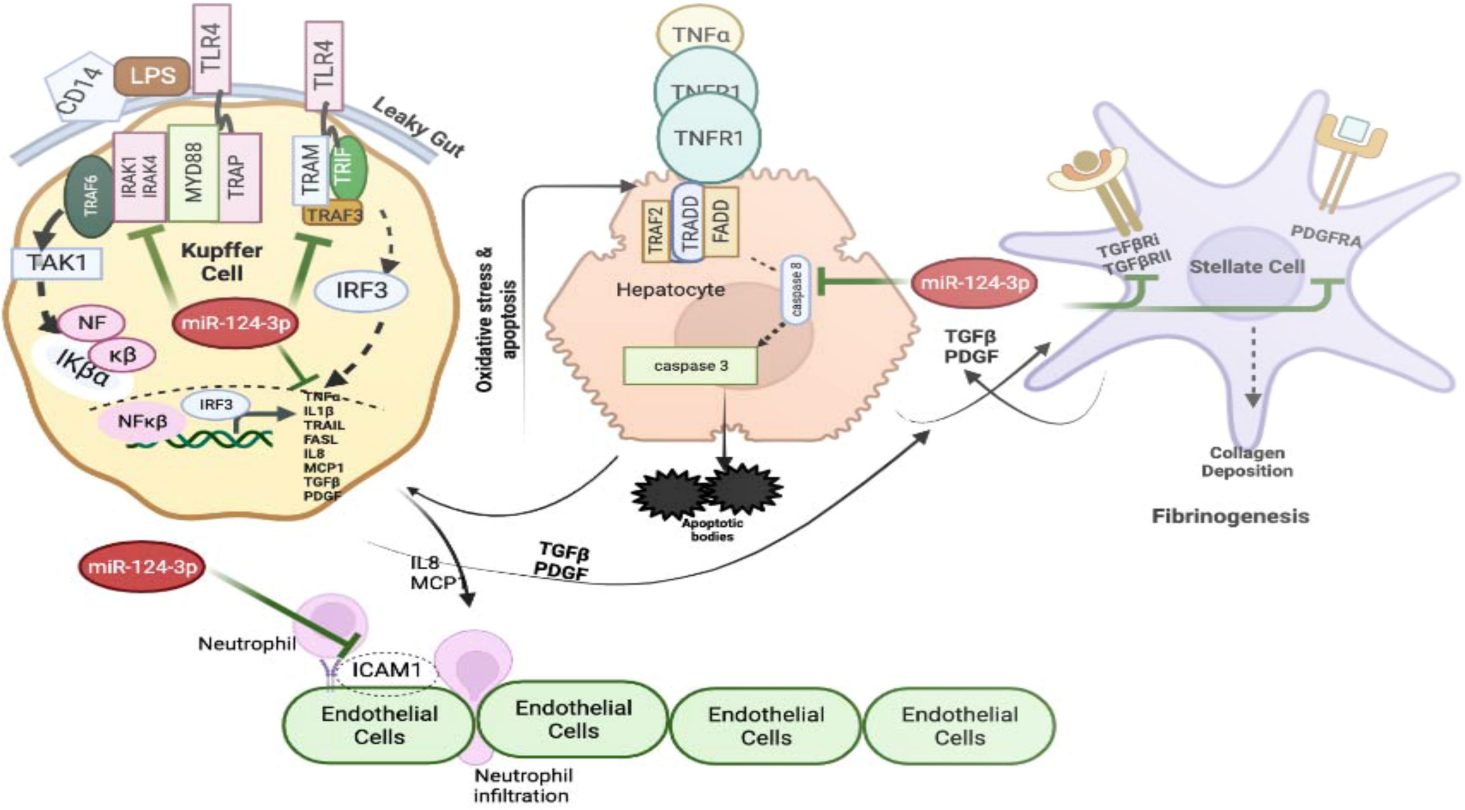
The graphical model shows the impact of increased level of TNF-α and IL1β on various cells present in the liver milieu and the therapeutic potential of miR-124-3p. The figure was generated using BioRender.com.

Cumulatively, these data suggest that alcohol sensitizes liver macrophages to secrete TNF-α and IL1β in liver microenvironment. Failure to control such situation triggers vicious activation of downstream cytokine cascade through MyD88 dependent and independent pathways leading to hepatocyte death, stellate cell activation and neutrophil infiltration. This causes progression of fibrosis and cirrhosis. Restoration of miR-124-3p in this milieu may resist the intercellular communications and withheld multiple downstream activation pathways suggesting its robust therapeutic potential for inflammatory diseases including ALD (**Figure 7**).

## Discussion

This study has presented several important findings. Firstly, a genetic study was conducted to determine the factors responsible for the susceptibility of only 10-30% of chronic alcoholics to the development of ALD and a novel approach was employed in the search of the risk factors for ALD. Unlike other association study, GSE143318 dataset, plasma cytokine profiling and a tissue qRT-PCR array of immune genes were used and only differentially expressed cytokines with high fold alteration both in the liver tissues and serum of the ALD patients were considered for this study. Among the six overexpressed cytokines, only two sequence variations were noted in the promoter region of TNF-α (rs361525G/A) and IL1β (rs1143627C/T) genes with higher frequency among ALD patients than ALC. Enrichment of TNF-α and IL1β in the liver microenvironment is associated with these variations independently. However, several *in vitro* assays were performed to comprehend the mechanism of development of ALD upon overproduction of TNF-α and IL1β. Lastly, a novel bioinformatics approach was employed to identify a common miRNA, which not only targeted TNF-α and IL1β genes but also several other cytokines, receptors and cargo molecules involved in LPS mediated TLR4 inflammatory signaling pathways. Binding of the miRNA to each of the target was verified by series of *in vitro* assays.

Unlike other complex genetic diseases, chronic ethanol drinkers are not always susceptible to develop ALD, although steatosis or fatty liver is commonly observed. Only one-third of the chronic drinkers develop steatohepatitis. Hence, the complex interplay between environment and host genetic makeup is the likelihood of ethanol dependence and also disease development. In this regard, numerous case-control and genome-wide association studies (GWAS) have identified and independently confirmed association of polymorphism(s) in myriad of genes including ethanol metabolism (ADH, ALDH, CYP2E1) (14, 15), oxidative stress response (GSTT1, GSTM1, MnSOD) (18), immune response (TNF-α, IL1β, IL10, CD14, TLR4, CTLA4) (19, 20), and fibrosis associated factors (TGFβ, MMP3) (19). Several issues such as low sample size, single centered study, inappropriate characterization of samples, no replication in an independent cohort and most importantly lack of functional characterization in disease developmental pathways have limited the authentication of the identified SNPs. To overcome these limitations, the preliminary risk factor association study was designed with six differentially expressed immune genes, which were overexpressed in liver tissues and also higher level was noted in the serum of the ALD patients suggesting alteration of these genes could be the predictor of the disease. Subsequently case-control association study revealed rs361525 (G/A) at -238 of TNF-α and rs1143627(C/T) at -31 in IL1β were significantly associated with ALD as observed in discovery cohort of 322 patients, validation cohort of 100 patients and 50 patients in a non-linked NASH group. Hence, these three tiers of verification enhance the degree of positive correlation of those two SNPs with the disease. Furthermore, comprehensive functional validation of each SNP in the disease progression pathways has strengthened our study.

Here, we have found that a high level of TNF-α and IL1β in the serum of chronic alcoholic with either -238GA or -31CT genotype correlated well with their liver disease status. Interestingly, our data suggest healthy individuals with those two variations were having more TNF-α and IL1β in the serum indicating their susceptibility towards inflammatory diseases including ALD. NF-kβ is the major transcription factor (TF) that triggers inflammatory response upon alcohol exposure. Excess binding of this TF to the -238A allele in the TNF-α promoter triggered expression of this cytokine in ALD patients which led to unrestricted activation of downstream IL6, IL8, MCP1, ICAM1 etc. As a result of it, peripheral neutrophils infiltrated to the damage site through endothelial layers (40). Binding of TNF-α to TRADD also induced extrinsic apoptosis pathways and ROS mediated death of liver cells, which in turn stimulated production of TGFβ and PDGF that signals to stellate cells to secrete fibrogenic collagens and other extracellular matrix proteins (ECM).

Given that, miRNAs are very well studied as a critical player in immune suppression, several seminal studies have shown it as a novel anti-inflammatory therapeutic agent. For example, miR-146 targets key adaptor molecules TRAF6 and IRAK1 in TLR/NFκβ mediated immune response pathways and high expression of this miRNA has been reported in Psoriasis causing inflammatory disorder (41). Whereas miR-124 promotes microglial immune suppression by targeting TRAF6 and p65 subunit of NFκβ (42). It also reduces BCG induced immune responses by reducing the production of proinflammatory molecules NF-κβ/IL-6/TNF-α/IL-1α/IL-8/IL-12α/IFN-β as it attenuates TLR6/MyD88/TRAF6 in alveolar epithelial cells (31). Corticosteroid and TNF-α inhibitors are tested for the treatment of alcoholic hepatitis but the results are controversial (43, 44) as both the molecules inhibit liver regeneration (45) and enhance the rate of bacterial infection.

Interestingly, this study suggests that miR-124 could diminish various inflammatory responses that are characteristics of several inflammatory diseases including ALD by abolishing both MyD88 dependent and independent inflammatory pathways. It has myriads of targets in different cells present in liver microenvironment such as MYD88/TRAF6/TRAF3/TNF-α/IL1β/MCP1/IL8 mostly in Kupffer cells; TRADD/Caspase8 in hepatocytes; PDGFRA/TGFBR2 on Stellate cells and ICAM1 on endothelial cells. *In vitro* co-culture assays had confirmed the robust potential of miR-124 in controlling the intercellular crosstalk executed during initiation and progression of ALD. The CM of THP1 with restored miR-124 expression and with low TNF-α and IL1β showed reduced level of apoptosis and ER stress in HepG2 cells, inhibited neutrophil infiltration through endothelial layers and reduced activation of Stellate cells followed by restricted fibrosis. Thus, by maintaining intracellular protein homeostasis, miRNA targeting multiple signaling proteins shared in diverse cellular pathways and serves as an efficient means to control robust signal activation or over activation of pro-inflammatory response. A recent study showed that depletion of neutrophil specific miR-223 protects mice from ALD (46) as it attenuates several macrophages associated with chemokines and cytokines such as TNF-α/MCP-1/MIP-1β/MIP-2 by downregulating p47^phox^.

Cumulatively, our clinical data and comprehensive functional analysis revealed that SNPs, rs361525 (G/A) at -238 in TNF-α and rs1143627(C/T) at -31 in IL1β are significantly associated with ALD. Upon exposure to alcohol, risk allele carrying individuals are having activation of LPS/CD14 mediated TLR4 signaling pathways which lead to overproduction of proinflammatory cytokines. These are the key players in the pathogenesis of ALD. Hence, these SNPs are two independent predictors for ALD among East Indian population and could be used in clinical practice to identify the susceptible individual who may develop ALD in future. Furthermore, *in vitro* assays and clinical data suggest that miR-124 is a potent immune modulator which remains unaltered between ALD and ALC individuals. But, exogenous overexpression of this miRNA could restrict multiple inflammatory cascades in ALD simultaneously and thus exhibit protective role in the disease management. Indeed liver-specific delivery of miRNA in human may be a challenge in order to generate better therapeutics for ALD but it is now an emerging field with a lot of hopes. Therapy with miRNA can achieve earlier unreachable medical benefits compared to conventional mono targeted therapeutics.

## Data availability statement

The raw data supporting the conclusions of this article will be made available by the authors, without undue reservation.

## Ethics statement

The studies involving humans were approved by Institute of Post Graduate Medical Education and Research, Kolkata, India (No: INST/ISE/1633) and Agartala Government Medical College, Tripura, India [No: F.4(5-2)/AGMC/Academic/Project/Research/2007/Sub-II.5649-52). The studies were conducted in accordance with the local legislation and institutional requirements. Written informed consent for participation in this study was provided by the participants’ legal guardians/next of kin.

## Author contributions

DD and SoB conceived and designed the study. DD collected biological samples from patients and executed genotyping, ELISA, qRT-PCR, and multiple assays in HepG2. SG did Bioplex chemokine screening and Co-culture experiments. SC, SuD, and NR helped in sample collection and genotyping. MS and AM did the FACS analysis and SM & ID performed the neutrophil migration assay. Patients were selected by PB & AC. ALD and normal liver tissue was provided by SA and SR. SaB generated the graphical abstract and SD read the manuscript carefully. All authors contributed to the article and approved the submitted version.
