# Supplementary figures and images for: Influence of polymorphisms in TNF-α and IL1β on susceptibility to alcohol induced liver diseases and therapeutic potential of miR-124-3p impeding TNF-α/IL1β mediated multi-cellular signaling in liver microenvironment
Source: Front Immunol. 2023 Dec 11;14:1241755. doi: 10.3389/fimmu.2023.1241755 (PMC10749309; doi:10.3389/fimmu.2023.1241755)

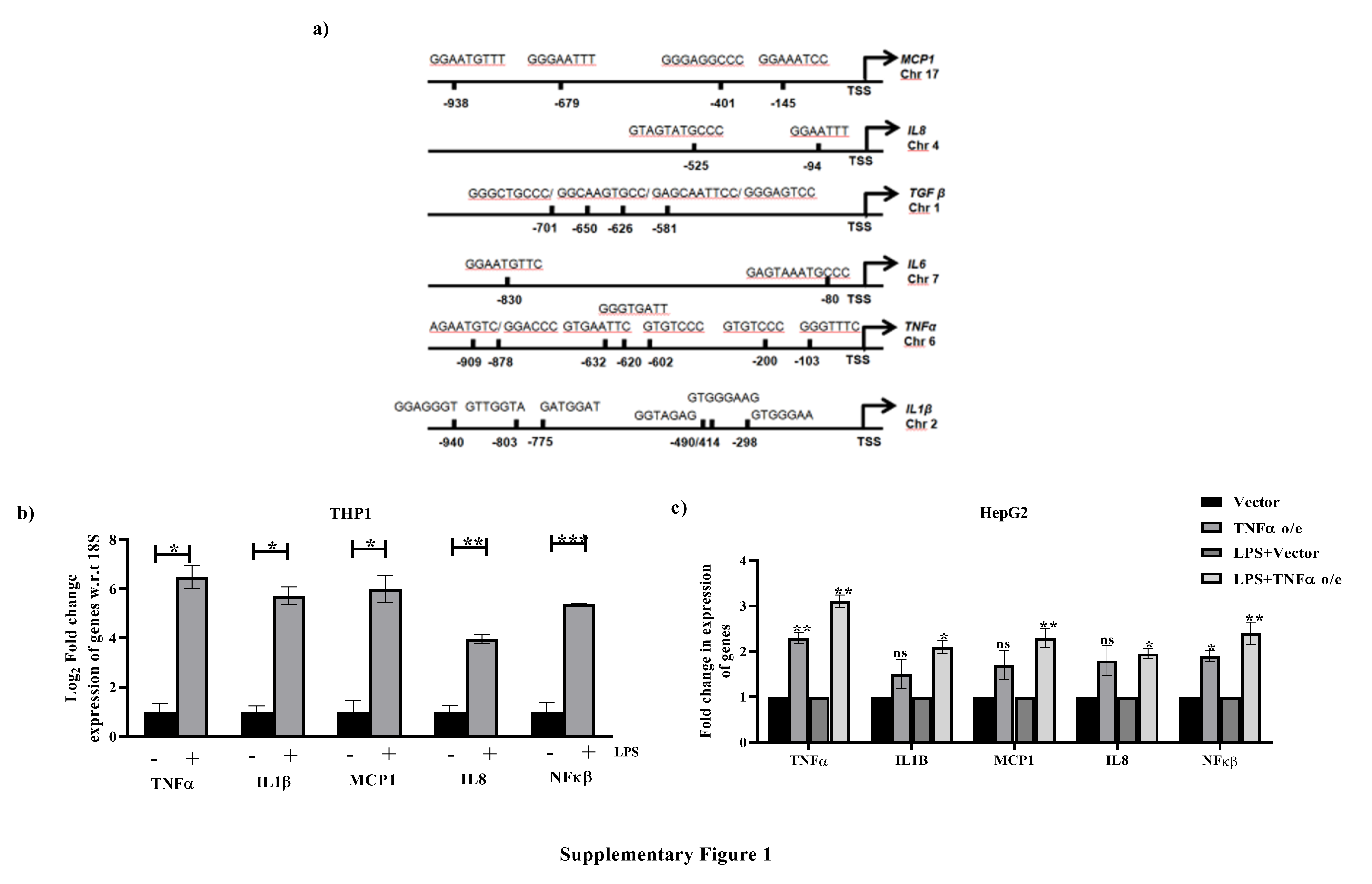

Supplement: Supplementary file 1 [file Image_1.tif]
