# Supplementary material for: Influence of polymorphisms in TNF-α and IL1β on susceptibility to alcohol induced liver diseases and therapeutic potential of miR-124-3p impeding TNF-α/IL1β mediated multi-cellular signaling in liver microenvironment
Source: Front Immunol. 2023 Dec 11;14:1241755. doi: 10.3389/fimmu.2023.1241755 (PMC10749309; doi:10.3389/fimmu.2023.1241755)
Supplement: Supplementary file 2 [file Table_1.docx]

| **Variables** | **ALD Patients**  **(n=100)** | **NASH Patients**  **(n=50)** |
| --- | --- | --- |
| **Epidemiology**  Gender M:F | 100:0 | 100:0 |
| Age, Years  (Mean±SD) | 45.16±7.789 | 40±9.148 |
| BMI (kg/m^2^)  (Mean±SD) | 20.37±5.06 | 28.31±4.137 |
| Ascitis | 40/100 | 0/50 |
| **Laboratory Results**  Albumin(g/dl)  (Mean±SD) | 3.43±0.73 | 4.325±0.3 |
| ALT(u/l)  (Median, Range) | 47.00  (20-179) | 43.50  (16-263) |
| AST(u/l)  (Median±SD) | 49.58  (21-220) | 66.00  (18-329) |
| ALP (IU/l)  (Mean±SD) | 178  (70-242) | 150  (88-190) |

**Supplementary Table 1:** Demographic, biochemical and clinical features of the validation cohort consist of ALD and NASH patients.

**Supplementary Table 2a :** Primers used for expression analysis

| **Gene name** | **Primer Sequence (5’-3’)** |
| --- | --- |
| TNF-α | GTGCTCCTCACCCACACCAT  GATTGATCTCAGCGCTGAGTC |
| IL1β | CCTGTCCTGCGTGTTGAAAGA  GGGAACTGGGCAGACTCAAA |
| IL 17 | AAACAACGATGACTCCTGGG  GAGGACCTTTTGGGATTGGT |
| IL 6 | AAAGAGGCACTGGCAGAAAA  AGCTCTGGCTTGTTCCTCAC |
| IL 8 | GCTGGCCGTGGCTCTCTTGG  GACAGAGCTCTCTTCCATCAG |
| IL 10 | AGATCTCCGAGATGCCTTCA  ATTCTTCACCTGCTCCACGG |
| IL 22 | TTGAGGTGTCCAACTTCCAGCA  AGCCGGACGTCTGTGTTGTTA |
| MCP1 | CCCCAGTCACCTGCTGTTAT  GCTGCAGATTCTTGGTTGT |
| IFNγ | ACTGACTTGAATGTCCAACGCA  ATCTGACTCCTTTTTCGCTTCC |
| TGFβ | TTCAAGCAGAGTACACACAGCATA  ACTCCGGTGACATCAAAAGATAAC |

**Supplementary Table 2b :** Primers used for 3’UTR cloning

| **Gene name** | **Primer Sequence (5’-3’)** |
| --- | --- |
| TFN α | ACTATCTCGACTTTGCCGAGTCT  AGACCATGTTTCTTTTCTAAAGCA |
| IL1β | TGTCTTCCTAAAGAGAGCTGTAC  TCCAAGACAAGTCAATGATCAGG |
| IL6 | GACAGGCTCAAAGACAGTGT  CCAAGCCTGACCAGCATCAC |
| IL8 | AGATGCAATACAAGATTCCTG  TCATTATTCCGTAATTCAACAC |
| MCP1 | TCCGAAGACTTGAACACTCACT  ACTGGAAGTCAGGTTTTGTACA |
| TGFβ | CATCGTGTACTACGTGGGCCGCAAG  TCTCAGAGTGTTGCTATGGTGAC |
| PDGFRA | GCTCGAGGCTGTGAGCCTTGCATGACATCA  GGCGGCCGCGGATGACAAGAGCAAAACTCCG |

**Supplementary Table 2c :** Primers used for mutagenesis

| **Gene name** | **Primer sequence (5’- 3’)** |
| --- | --- |
| TFN α | GCCCCCTGGCCTCTGAGGCATCTTTTGATTATGT  TACATGGGAACAGCCTATTGTTCAGCTCCGTTTT |
| IL1β | ATGCCCAACTGCCTCCGTAAGGGTAGTGCTAAGA  TGGTGTAGACAACAGGAAAGTCCAGGCTATAGCC |
| IL8 | GTCCTTGTTCCACTGAGGCATGGTTTCTCCTTTA  TTGTGGATCCTGGCTACGAGACTAGGGTTGCCAG |
| PDFFRA | GACAAGCTGTATCACTCCGTACGTTTATATTTTT  ATAAATGTTTTGGCAGTATTCTCCAAGTCTATAT |

**Supplementary Table 3:** Binding of miR-124-3p to the 3’UTR sequences of the target genes

| **Gene name** | **Seed sequence Binding Sites** |
| --- | --- |
| MYD88 | 3' CCGUAAGUGGCGC**ACGGAA**U 5' miR-124  \| \| \| \| \| \| \| \|  5' UUUGUACCUUGAU**UGCCUU**A 3' MYD88 |
| TRAF6 | 3' CCGUAAGUGGCGC**ACGGAA**U 5' miR-124  \| \| \| \| \| \| \|  5'CCUGGAGAAAACAG**UGCCUU**U 3’TRAF6 |
| MCP1 | 3' CCGUAAGUGGCGC**ACGGAA**U 5' miR-124  \| \| \| \| \| \| \|  5'AUGUGAAACAUUA**UGCCUU**A 3' MCP1 |
| IL8 | 3'CCGUAAGUGGCGC**ACGGAA**U 5' miR-124  \| \| \| \| \| \| \|  5' CUUGUUC-CACUG**UGCCUU**G 3' IL8 |
| TRAF3 | 3’CCGUAAGUGGCGC**ACGGAA**U 5' miR-124  \| \| \| \| \| \| \| \| \|  5’CAUACGGCCCACG**UGCCUU**A 3’ TRAF3 |
| TRADD | 3' CCGUAAGUGGCGC**ACGGAA**U 5' miR-124  \| \| \| \| \| \|  5' GGGCAGAGUUGAU**UGCCUU**C 3' TRADD |
| Caspase8 | 3'CCGUAAGUGGCGC**ACGGAA**U 5' miR-124  \| \| \| \| \| \|  5'CAAACUUGCUUUA**UGCCUU**C 3 ' CASP8 |
| PDGFRA | 3' CCGUAAGUGGCGC**ACGGAA**U 5' miR-124  \| \| \| \| \| \|  5'CAAGCUGUAUCAC**UGCCUU**C 3' PDGFRA |
| TGFβR2 | 3' CCGUAAGUGGCGC**ACGGAA**U 5' miR-124  \| \| \| \| \| \|  5' AUAGAGCAUUCUA**UGCCUU**U 3' TGFΒR2 |
| ICAM1 | 3’CCGUAAGUGG-CGC**ACGGAA**U 5' miR-124  \| \| \| \| \| \| \| \| \|  5'CUCAUUGGCCAACC**UGCCUU**U 3' ICAM1 |
